# Quality control on the frontier

**DOI:** 10.3389/fgene.2014.00157

**Published:** 2014-05-27

**Authors:** Konrad H. Paszkiewicz, Audrey Farbos, Paul O'Neill, Karen Moore

**Affiliations:** Exeter Sequencing Service, Biosciences, College of Life and Environmental Science, University of ExeterExeter, UK

**Keywords:** sequencing, Illumina, quality control, core-facility, best practice

## Abstract

In the world of high-throughput sequencing there are numerous challenges to effective data quality control. There are no single quality metrics which are appropriate in all conditions. Here we detail the different open source software used at the Exeter Sequencing Service to provide generic quality control information, as well as more specific metrics for genomic and transcriptomic libraries run on Illumina platforms.

## Introduction

High-throughput production in any field requires quality metrics. Often it is motivated by the need to ensure that clients and downstream users obtain products in good condition. However, it is also used to monitor processes and improve them. In the context of DNA sequencing, the challenges posed by the high sample throughput of a single instrument necessitate the generation of informative quality metrics. These challenges include evaluation of sample input, library quality and whether project requirements can be met using the data generated from a given set of results.

The accessibility of high-throughput sequencing instruments means that sequencing itself will eventually become as ubiquitous as simple PCR. However, in much the same way as a conceptually simple operation is subject to a myriad of parameters, sequencing is an imperfect process subject to many biases. The role of a good sequencing service provider is to identify any such biases, correct where possible and highlight potential downstream impacts to those interpreting the data.

The Exeter Sequencing Service has been operating Illumina (San Diego, CA) sequencing platforms since 2008. It is a small to mid-size sequencing academic core-facility which today operates MiSeq and HiSeq instruments. It is the type of facility which often operates within limited financial constraints and whose staff are heavily relied upon to provide expert advice to researchers. It is often the case that such facilities are heavily reliant on existing tools produced by the community to generate informative quality control data. As such, they are often test-beds for new tools/techniques within the institution.

## Wet lab quality control

Prior to samples being received by the facility, all projects are discussed to evaluate requirements and to determine which methodologies are likely to provide the best value for the analysis at hand.

DNA or RNA samples are received from users using a custom LIMS system to capture a number of metrics and metadata regarding samples. This satisfies the requirements of public metagenomics, transcriptomics, and genomics databases. Many of these standards are developed and published by the Genomic Standards Consortium (GSC) (Field et al., 2008) or form part of the National Center for Biotechnology Information databases (Edgar, 2002; Wheeler et al., 2008). Most importantly, it enables the facility to insist on various quality control checks to be submitted for evaluation prior to sample receipt. Any issues with poor quality samples can be detected prior to any sequencing or library preparation cost being incurred by the downstream user.

### Assessing the quality of nucleic acids prior to library preparation and illumina sequencing

Fluorescent dyes that intercalate between bases of nucleic acids are used as a basis for quantification of nucleic acids and, in conjunction with gel electrophoresis, to determine the size of the molecules resolved, and therefore make judgments about the quality of the isolated DNA or RNA. By using specific dyes for DNA and RNA that have very low fluorescence until they bind the target molecule it is possible to accurately determine the concentration of each type of molecule in a mixture even if other biomolecules are present. This results in more precise quantification than UV absorbance methods which are not selective. Qubit assays (Life Technologies) uses the Qubit fluorometer for quantification, whereas the Pico Green assay (Life Technologies) uses a microplate reader to determine fluorescence in a liquid assays.

Nucleic acids separated by fluorescent agarose gel electrophoresis provide the simplest method for assessing the quality where the concentration of DNA is sufficiently high. The agarose gel image should provide information about the quality of the DNA sample indicating the ratio of degraded DNA to high molecular weight DNA.

Assays that use fluorescent dyes in conjunction with microfluidic electrophoresis include Bioanalyser (Agilent), Labchip GX (Perkin Elmer), QIAxcel (QIAGEN), and Fragment Analyser (VH bio ltd); these instruments can be used to analyse dsDNA fragments, RNA or prepared NGS libraries where material is precious.

UV absorbance ratios at 230:260 nm and 260:280 nm can provide additional information regarding purity of the sample, in particular, presence of phenol which absorbs with a peak at 270 nm, can contribute to the over-estimation of DNA concentration, whereas, humic acids that may be present in DNA isolated from soil absorb at 230 nm, as do phelolate ions and thiocyanates that may be used to isolate RNA.

In general pure DNA, A260/280 is ~1.8 when measured in 10 mM TrisHCl pH7.5, and for pure RNA A260/280 is ~2 when measured in water. Chitin is a structural polysaccharide that is a major component of the carapaces, crusts and shells of crustaceans such as shrimps, crabs and lobsters; it is also an ingredient of cell walls in fungi and yeast which may bind to DNA and impact on library preparation, possibly by artificially depressing DNA concentration (Kumirska et al., 2010; Azofeifa et al., 2012). RNA contamination may inhibit some downstream steps. When RNA contamination is evident treatment with DNase-free RNase I is a simple remedy.

### DNA submission

For DNA fragment library preparation fragmentation should be as random as possible therefore high molecular weight DNA is required. In circumstances where only degraded DNA is available library preparation may be less efficient which may require greater sequencing coverage to enable genome assembly.

### RNA submission

**Figure 1:**
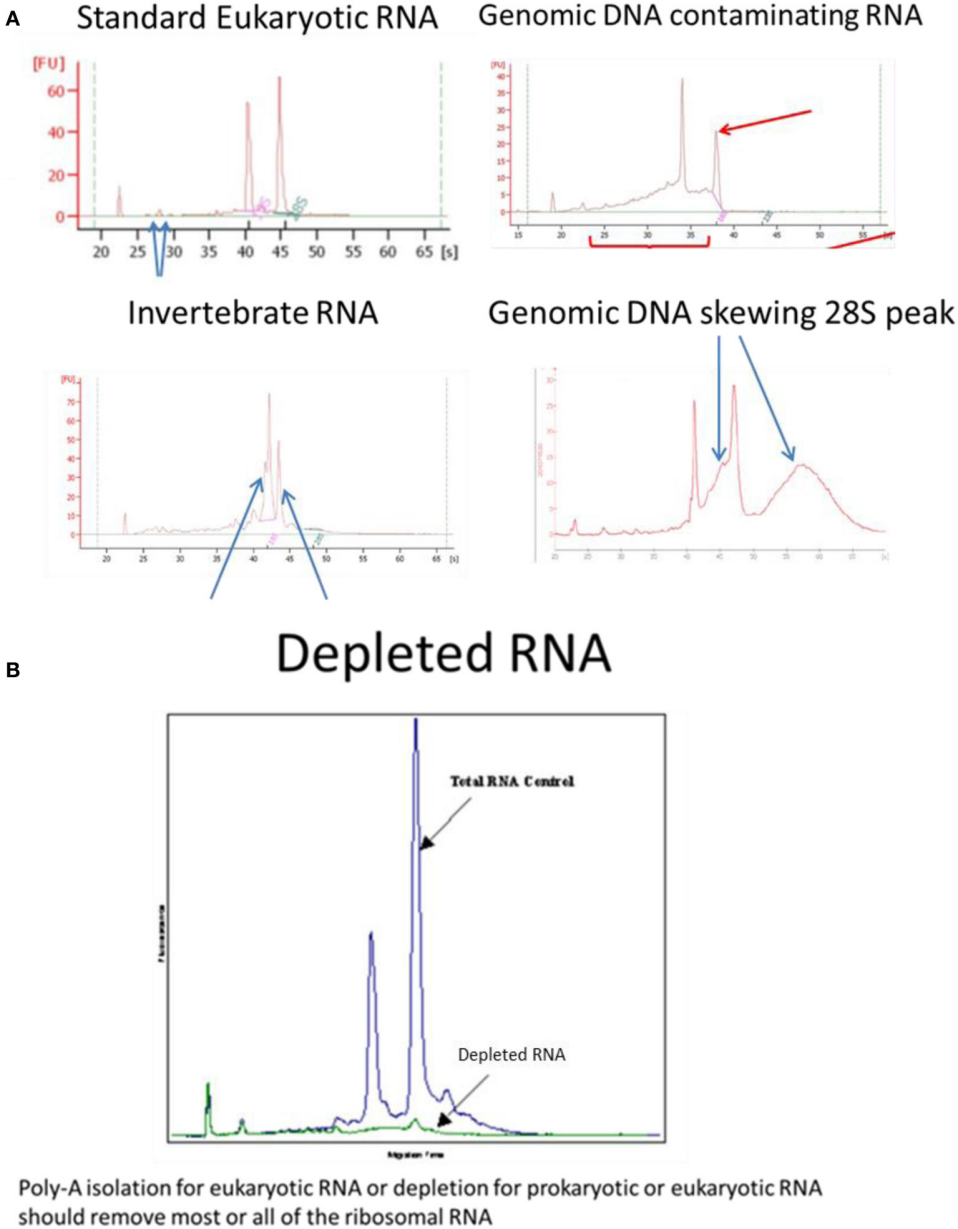
**Examples of Bioanalyser assay interpretation for a variety of RNAs. (A)** Standard Eukaryotic RNA shows a 28S rRNA band at 4.5 kb that should be twice the intensity of the 18S rRNA band at 1.9 kb (human) resulting in a RIN = 8.0–10.0. Small peaks are sometimes present after the marker that represent 5S and 5.8S subunits, tRNAs and small RNA fragments about 100 bp; these are more obvious when using phenol or trizol exterection methods, QIagen columns will generally remove small RNAs. When degraded 28S RNA is reduced and more fragments are detected around the 18S RNA subunit resulting in RIN = 6.4, which is below the quality required for high throughput DNA sequencing. Invertebrate RNA results in fragmentation of the 28S rRNA into two bands that co-migrate with the 18S rRNA resulting in aberrant RIN score of <8.0 although the mRNA is unaffected and suitable for sequencing. Genomic DNA can skew the 28S RNA peak but can easily be remedied by RNAse-free DNase1 digestion. **(B)** Ribosomal RNA removal by isolation of poly-A-RNA assessed by Bioanalyser RNA assay.

For RNA samples, microfluidic electrophoresis instruments provide an electropherogram and a measure of RNA integrity such as RIN (Agilent), RIS (QIAGEN) RQS (Perkin Elmer) RQN (VH bio) calculated by the software based on the entire electrophoretic trace of the RNA sample including the presence or absence of degradation products. The RNA quality/integrity score is independent of sample concentration, instrument and analyst therefore provides a standard for vertebrate, plant or bacterial RNA integrity (Mueller et al., 2004; Imbeaud et al., 2005; Schroeder et al., 2006). One drawback with the RIN assay is highlighted for samples where the ribosomal RNA subunits behave differently to standard “vertebrate” RNA, for example, the 28S rRNA subunit of most insects and a number of species of crustacean consist of two separate fragments that are hydrogen bonded together; depending on pre-treatment and electrophoresis conditions, disruption of these hydrogen bonds occurs and the two fragments co-migrate with the 18S rRNA (Winnebeck et al., 2010) resulting in irregular or meaningless RIN scores. DNA contamination of RNA can be observed in traces around the 28S RNA peak which is remedied by RNAse-free DNAse1 digestion followed by re-purification of the RNA to remove the enzyme and buffer rather than heat denaturation of the enzyme which risks degradation of the RNA and retention of the enzyme buffer. The effectiveness of poly-A isolation or ribosomal RNA depletion, used to enrich for mRNA, can be confirmed or compared using the bioanalyser (Figure 1).

### Other considerations for library preparation

The importance of sample quality before library preparation is emphasized to users of the service however occasionally libraries may be prepared from poorer quality material because no other material is available.

For cases where GC bias is expected and PCR is required as part of library preparation, caution must be exercised in the choice of polymerase (Aird et al., 2011; Ross et al., 2013). Typically we use Kappa HiFi polymerase for most genomic libraries requiring PCR (Quai et al., 2012).

**Figure 2:**
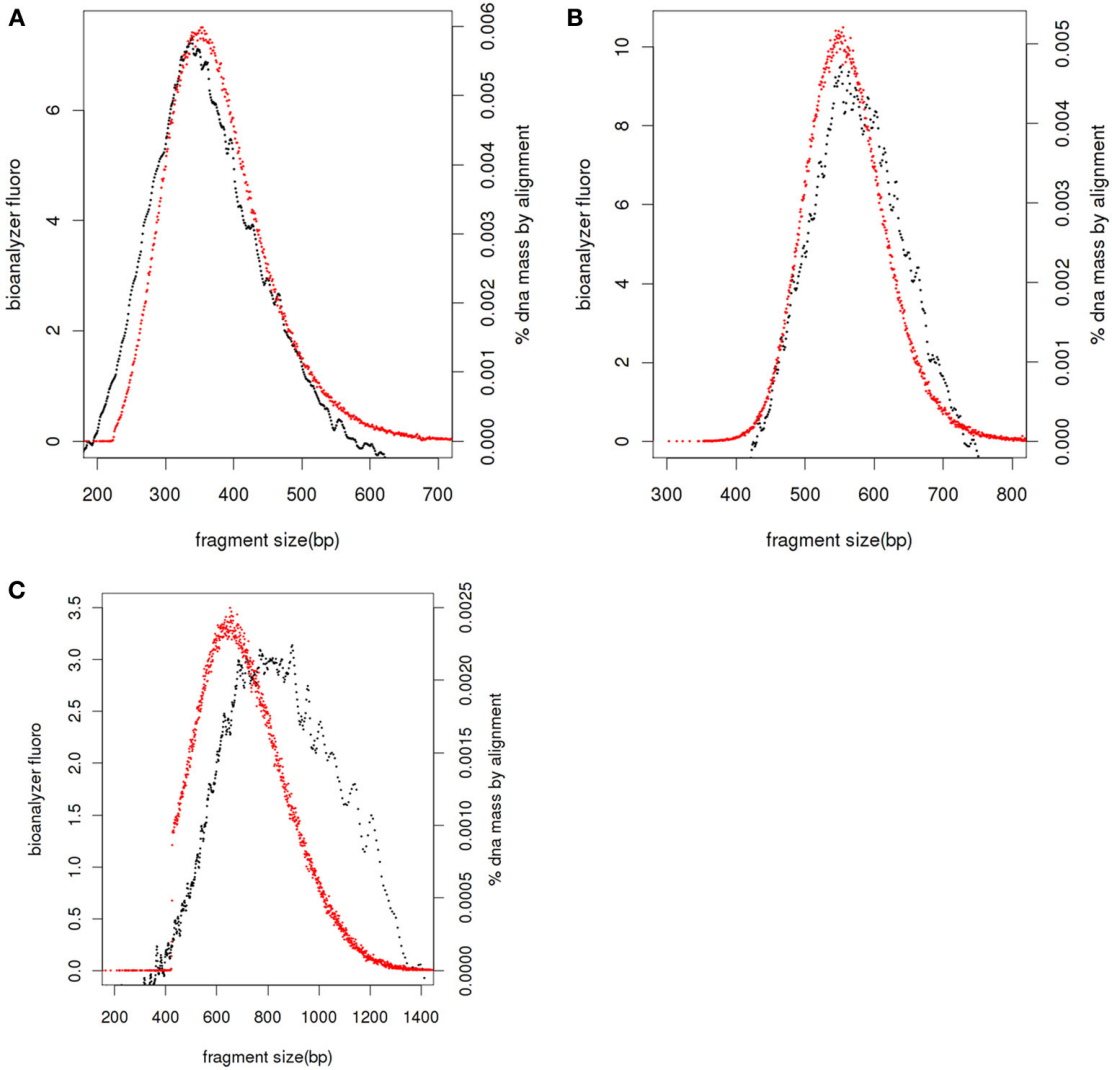
**Library fragment size distribution**. Bioanalyser fluorescence values (black), realignment of paired end reads against reference genome or *de novo* assembly and adjusted by 126 bases to account for adapters (red) for libraries with average sizes of 360 bases **(A)**, 550 bases **(B)**, and 810 bases **(C)**.

If service users have prepared sequencing libraries themselves we ask for the same QC of the final libraries as we would undertake if libraries had been prepared by Exeter Sequencing Service, including Bioanalyser DNA traces and/or qPCR quantitation. Bioanalyser DNA assays allow the size distribution of the final library to be determined together with presence of any remaining adapter-dimers. The size of fragments in the library includes the insert DNA for sequencing and adapters sequences which, for standard libraries, add 126 bases. After sequencing, the distance between the paired-end reads can be compared to the fragment sizes for the library (Figure 2); libraries with small inserts clustering is efficient for all molecules sizes (Figure 2A) whereas as fragment sizes increase clustering is more efficient for smaller fragments (Figures 2B,C) leading to a shift to the left in the paired end read distance relative to the Bioanalyser trace.

Once accepted by the facility all samples are assigned project and sample identifiers. When necessary qPCR or MiSeq nano runs are undertaken to determine optimum loading concentrations.

## Data management

The Illumina Genome Analyser, HiSeq and MiSeq instruments generate basecall data during a run using Illumina's RTA software. To reduce time spent during data transfer and to ensure maximum uptime, we connect each of our HiSeq 2500 instruments over a dedicated 1Gbit Ethernet link to a separate Dell R510 server each with 60Tb attached MD3xxx storage. The lower data volumes produced by the MiSeq instrument means that it is possible to connect such instruments to a single server over a shared 1 gigabit Ethernet link.

The Illumina bcl2fastq package is used to convert the proprietary Illumina BCL files to Sanger fastq format and demultiplex samples based on the information provided in the standard Illumina-formatted sample sheet. A simple perl script ensures that the sample sheet is in the correct format prior to initiating the demultiplexing.

Once complete a series of generic quality control metrics are generated using open source programs (see below). The results of these are collated into a summary html-formatted file. After these steps are completed the data for each project is copied to a compute cluster which shares storage with an FTP server. FASTQ data is archived after 6–9 months to Amazon Glacier (Seattle, WA) unless otherwise requested.

## Data transfer

Once generic data QC and any subsequent analysis is completed, data is delivered to users via an FTP server. We use pure-ftp for the purpose as it enables relatively straightforward auto-generation of FTP accounts and passwords (http://www.pureftpd.org). These are then emailed to users along with a guide to their data and instructions on how to access it. Many users are unfamiliar with FTP clients and terms such as “host,” “server” or applicable port numbers, so it is important to provide such instructions in simple language with screenshots to help guide the user.

MD5 checksums (Rivest, 1992) are strings of 32 characters produced by hash functions applied to files. A file with unique content should produce a unique checksum. These checksum values can be used to check whether files have been transferred with fidelity. It should be noted however that MD5 checksum collisions have been known to occur (i.e., two files with different contents producing the same checksum), however, the likelihood of this happening for a given file and a corruption or truncation of that same file is very low. We produce checksums for FASTQ files only. Due to their size and vulnerability to corruption, users are unlikely to notice a problem with their FASTQ files until they are some way into their analysis.

## Overview of available quality control tools

There are a wide variety of tools which are capable of generating QC metrics. These include FastQC (Andrews, 2010a,b), HTQC (Yang et al., 2013), NGS QC Toolkit (Patel and Jain, 2012), SolexaQA (Cox et al., 2010), Kraken (Davis et al., 2013), QC-Chain (Zhou et al., 2013).

Each tool has a different set of features available, FastQC focuses entirely on the calculation and visualization of quality metrics, and provide no facility to correct problems HTQC and SolexaQA are strong in this area and also provide some correction. These also include a tile based quality assessment best executed be SolexaQA not available in FastQC. The rest lean toward the trimming and filtering of reads. A summary of a selection of features can be seen in Table 1.

**Table 1:**
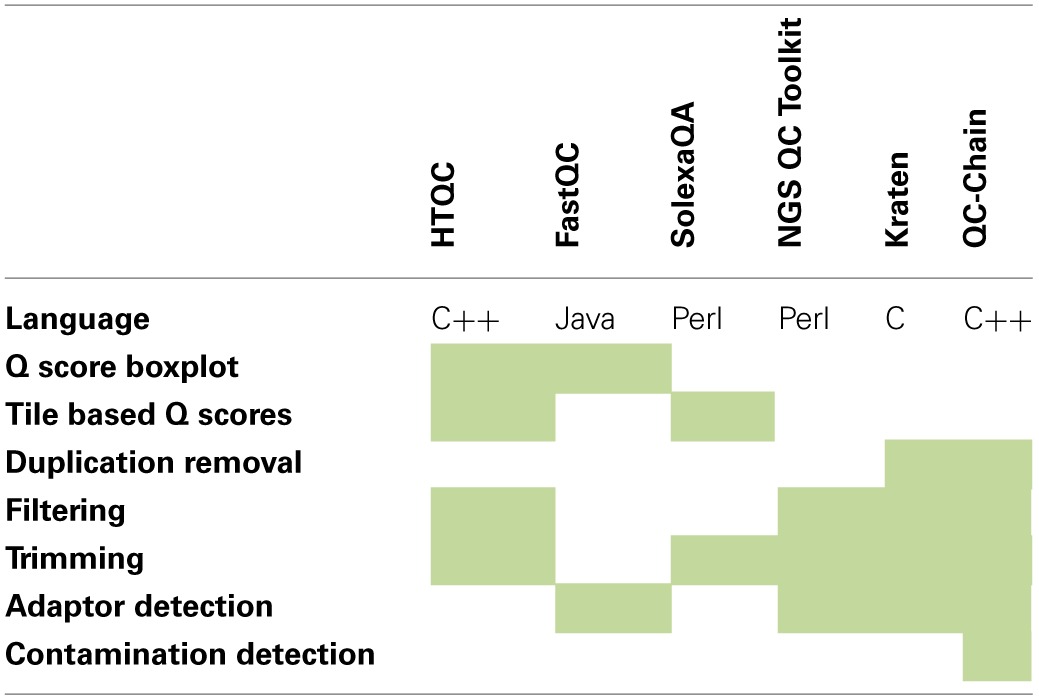
**Comparison of features in QC Toolkits**.

Comparison of feature of software packages for quality control of Illumina read data. Shaded areas indicate that the feature is present.

## Generic data quality control

Initial quality control is independent of any particular library type. These metrics include:

- Total numbers of reads generated per sample
- Quality score distribution across reads
- Base-call distribution across reads
- Quantification of any possible contaminants including adaptor sequences and primer-dimers
- Estimates of read duplication rates

**Figure 3:**
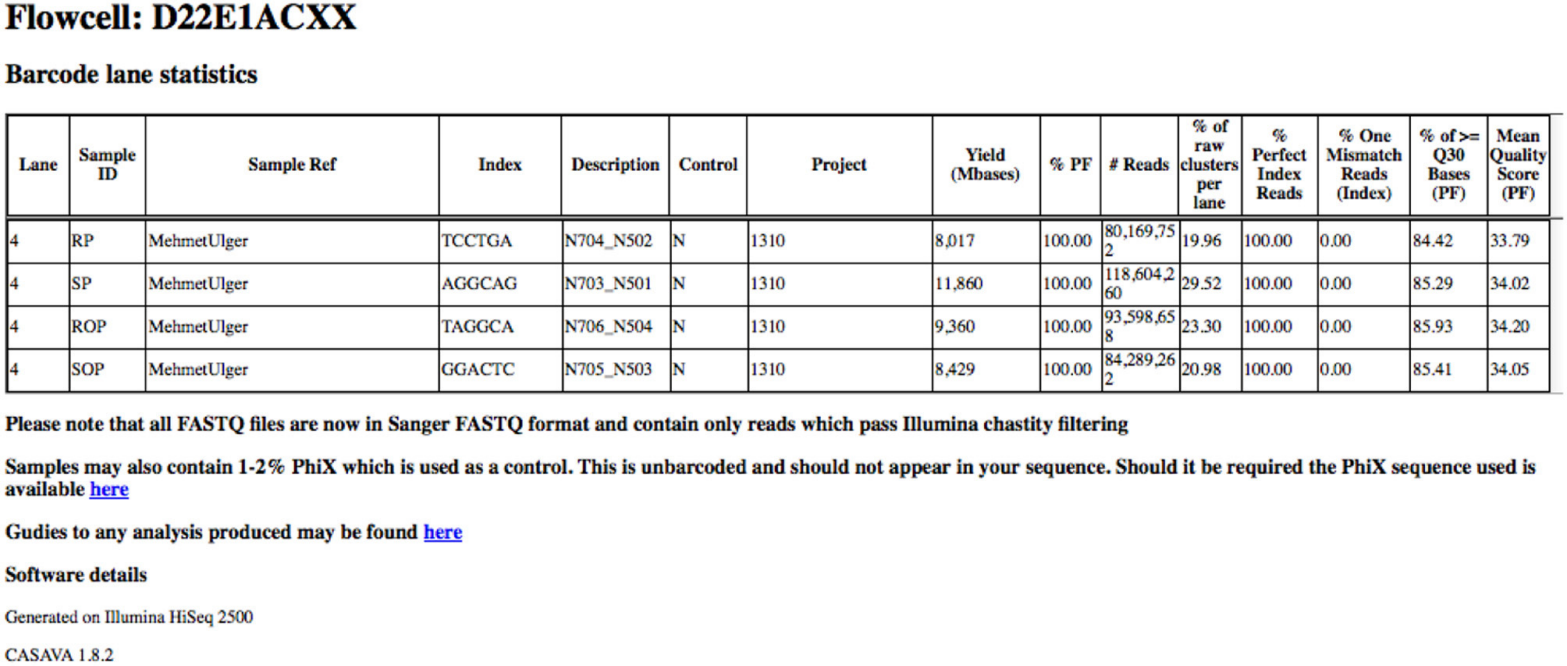
**Basic read metrics extracted on a per-project basis**. Basic read metrics extracted on a per-sample basis from the Illumina Demultiplex_stats.html file produced by the bcl2fastq pipeline. Additional information has also been added.

**Figure 4:**
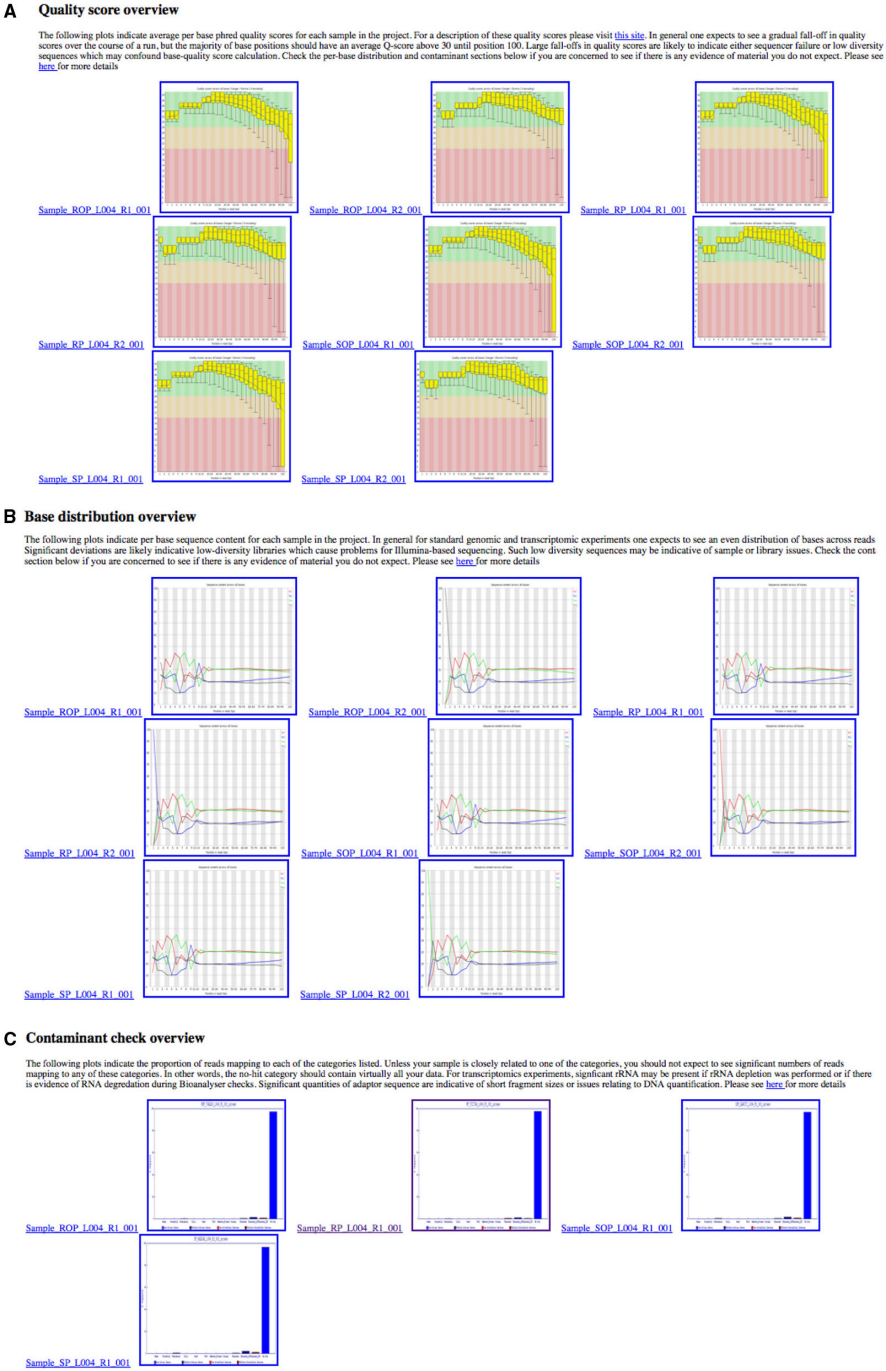
**Overview of quality control metrics across multiple samples in a project**. These plots are collated into a single HTML summary file for each project, making it easy to see any quality **(A)**, nucleotide **(B)**, or contaminant **(C)** issues at-a-glance.

In order to provide an overview of these metrics for all samples within a project, these metrics are collated into a single HTML summary overview file (Figure 3). To do this we process the Demultiplex_Stats.html file produced by the bcl2fastq pipeline. Only information specific to a particular project is retained. To obtain images for quality score and base-call distributions we use the FastQC program (Andrews, 2010a,b) for both read 1 and read 2. This ubiquitous tool provides a wide-variety of useful metrics in a user-friendly HTML format. The images themselves are stored separately in PNG format which can be easily extracted and re-packaged using custom scripts. We then extract plots relating to quality score and base-call distribution and base-call and include these in the overview summary file (Figures 4A,B).

**Figure 5:**
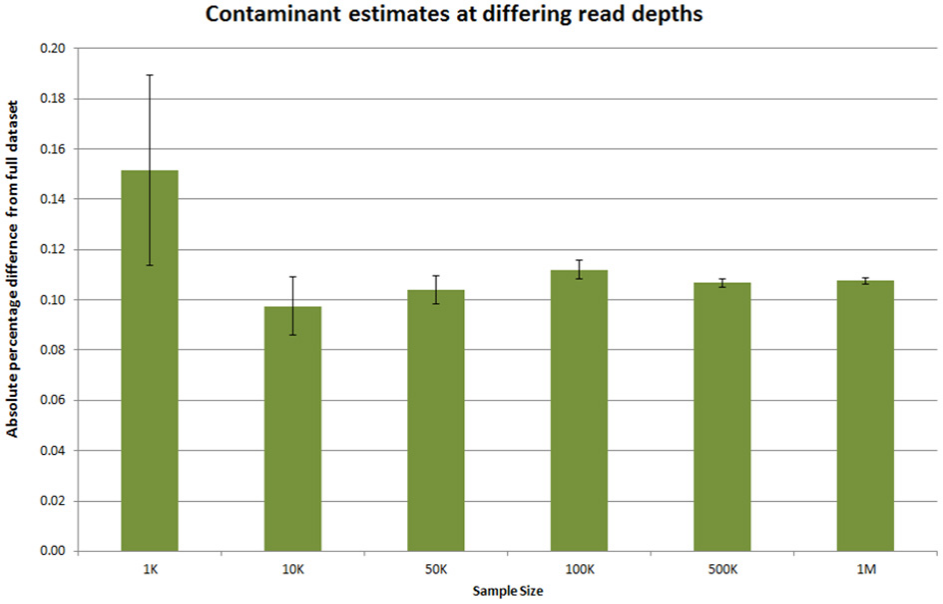
**Estimating required read sampling for contaminant checks**. Ten million reads from an Illumina RNA-seq dataset was subsampled at various numbers of reads. The number of rRNA contaminant reads in this dataset was 1.86% when calculated over the full dataset. The absolute percentage difference at different sub-sample sizes was calculated for 500 replicates at each depth and the average shown. The error bars indicate the 95% confidence interval for the absolute percentage difference.

Providing information on potential contaminants is also crucial, both for a facility and for the user. These contaminants can have a wide variety of sources and may be related to the original extraction, library preparation or index read barcode issues. To provide a visual representation of these estimates we use the fastq_screen tool (Andrews, 2010a,b) to subsample 500,000 reads from each sample in read 1. These are then aligned to genomic sequences *E.coli, M.Musculus, D.melanogaster, A.thaliana, H.sapiens, PhiX 174* and a non-redundant set of rRNA sequences from the Ribosomal Database Project (Wang et al., 2007) and a non-redundant set of viral sequences. The genomes were selected as they are among the most commonly sequenced at our facility. fastq_screen provides data in both textual and graphical png formats. The png plots are included in the overview summary file. Figure 4C illustrates the effect of *PhiX* contamination in a library. To demonstrate that this is sufficient to detect contamination as low as 1–2%, we used data from an RNA-seq experiment containing 10 million reads. An analysis of the full dataset showed a 1.86% level of rRNA contamination. We then sub-sampled the data at different numbers of sequences using 500 bootstrap replicates for each number of sequences. Figure 5 shows that as little as 1000 reads is sufficient to quantify the proportion of contaminating material. We routinely sample at larger sample sizes to ensure that in the presence of multiple contaminants and larger data volumes we are still able to provide confident estimates.

Duplication rates are also useful information for all library types. For genomic libraries, identical reads can indicate the presence of PCR-duplicates or fragmentation biases. These are uninformative for analysis and, essentially waste sequencing capacity. For transcriptomic or ChIP-libraries, the proportion of duplicated reads is less informative, but can be indicative of library complexity. Libraries dominated by a few transcripts or peaks will tend to have a higher proportion of duplicated reads. However, for transcriptomic libraries the RNA-SeqQC (DeLuca et al., 2012) pipeline described below is more informative. FastQC bases its calculation of duplication rates on the the first 50 bp of each read and the first 200,000 reads. Our users typically prefer estimates over a greater part of the read. Instead duplication rates are calculated using the FASTX-toolkit (Hannon, 2010) for each read 1 fastq file and the results collated accordingly. Estimating duplication rates in this way without remapping to a reference genome can lead to underestimates of duplication rates as sequencing errors prevent exact matches. However, as many genomes lack references and not all projects we undertake require us to perform de-novo assembly we have found this methodology to be a good compromise.

## Genomic sequencing quality checks

Some projects require remapping of one or more samples to a reference genome. In these cases there are a number of additional quality control metrics which can be generated. Typically we align against one or more reference genomes using the BWA package (Li and Durbin, 2009). If there are multiple library types present for a single sample, these can be merged into a single Binary AlignMent (BAM) file (Li et al., 2009) and can be tracked separately in a single BAM file.

To generate various statistics a PDF or HTML-formatted report is produced with the QualiMap package (García-Alcalde et al., 2012). These include: numbers of reads mapping to the reference genome; insert size distributions; coverage statistics; mapping quality; and a variety of plots to identify regions of the reference genome which may contain structural variants. This package also provides an alternative estimate of read duplicates which in most cases is more accurate than simply counting exactly matching reads.

**Figure 6:**
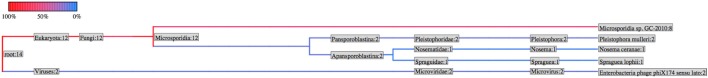
**Taxonomy of unmapped reads assembled into contigs**. A graphical representation of the number of contigs mapping to each level of the NCBI Taxonomy. The colors represent the number of contigs mapping to each branch.

An additional useful QC check is to perform a *de novo* assembly on any reads which do not map to the reference sequences. To do this we utilize the Velvet assembler together with the VelvetOptimizer package (Zerbino and Birney, 2008). The presence of the reference genome at a much higher relative abundance will often allow assemblers to remove most contaminant reads from the assembly by excluding low-abundance k-mers. However, this cannot always be relied upon and risks introducing contaminant genomes into published genome assemblies. By assembling only those reads which do not directly map onto one or more reference genomes, it is often much easier to spot contaminant genomes. After assembly, the resulting contigs can be searched against the NCBI non-redundant nucleotide database using the Megablast algorithm (Morgulis et al., 2008). The resulting output is then processed by the gi2taxonomy.py and t2ps_wrapper.py scripts (adapted from the Galaxy distribution Goecks et al., 2010). Figure 6 illustrates this in a microsporidian genome assembly where contaminating *PhiX* reads have resulted in *PhiX* contigs being generated. This is a clear warning to any downstream user that the data may need to be cleaned further prior to any de-novo assembly.

## RNA-seq quality checks

RNA-seq involves a number of additional steps during library preparation which can result in biases being introduced. These include the polyA extraction/ribosomal depletion steps, cDNA synthesis and PCR amplification (Hansen et al., 2010). Some parts of the generic quality control pipeline can provide indications of problems (e.g., rRNA contamination, low library diversity).

To ensure that the final library is a reasonable facsimile of the original RNA transcripts, we spike in 1% of the External RNA Control Consortium (ERCC) spike-in mix (Jiang et al., 2011) to the total RNA of each sample prior the library preparation. These are a set of 96 synthetic transcripts derived from bacterial genomes present at a variety of known abundances. As these are of a known sequence, we are able to use these to evaluate the success of an RNA preparation. (If sequencing bacterial transcriptomes, caution must be exercised to ensure none of the spike-in transcripts map to the bacterial species in the experiment.)

**Figure 7:**
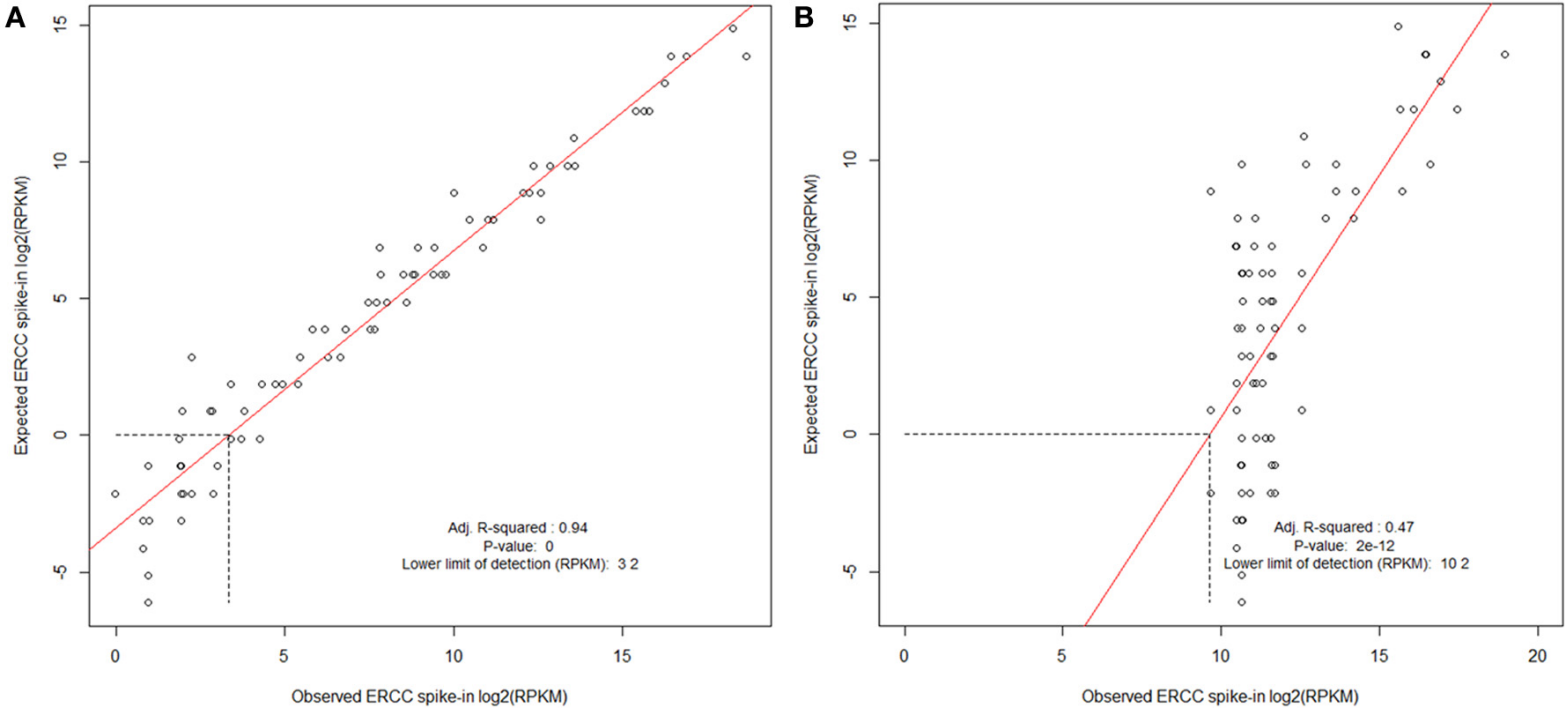
**Evaluating ERCC spike-in control results**. Examples of cDNA libraries prepared from *Arabidopsis thaliana* RNA and Mouse infected with *Burkholderia pseudomallei* RNA containing ERCC Spike-In. Spike-In mix was added to total RNA before preparation of the sequencing library. **(A)** *A. thaliana* poly-A RNA was isolated, and a sequencing library prepared using ScriptSeq v2 (Epicentre). **(B)** Trial *B. pseudomallei* sequencing library was prepared from RNA extracted from liver of a *B. pseudomallei* infected mouse; prokaryotic RNA was enriched using Microbe enrich kit (Invitrogen) and bacterial ribosomal RNA was reduced using MicrobeExpress (Invitrogen) before ScriptSeq v2 sequencing library preparation. ERCC spike in mix is polyadenlyated and the majority would be expected to have been removed during the library preparation resulting in a poor correlation and lower limit of detection, thereby contributing to the protocol development. Libraries were processed and sequenced on the Illumina HiSeq2500. The data were normalized to reads per kilobase of exon model per million mapped reads (RPKM) and filtered using a sensitivity threshold set arbitrarily at 1 RPKM (shown by the horizontal dotted line in at log2 RPKM = 0; Mortazavi et al., 2008).

To evaluate the success of an RNA preparation we map the full set of reads to the set of ERCC transcripts using the Bowtie package (Langmead et al., 2009). The number of reads mapping to each transcript are then extracted using the samtools idxstats package (Li et al., 2009) and RPKM values calculated (Mortazavi et al., 2008). These are then compared to the expected abundances and a log-log plot is produced. This enables the calculation of a lower-limit of detection for each sample and ensures that transcript abundance for the controls is consistent across the range of expression. Figure 7 illustrates this and shows the result of a “good” sample vs. a “bad” sample. As technology changes, it is our hope that such spike-in control data can be used to help compare samples between platforms. Reads which do not map to the ERCC transcripts can then go on to an RNA-seq analysis.

An excellent quality control package for RNA-seq data is the RNASeqQC package. Unfortunately, it has very particular requirements relating to the annotation format and thus can only be used with organisms with GTF-formatted annotation. Nonetheless, we find it to be a very valuable tool. This tool is used after the removal and evaluation of reads mapping to the ERCC reference transcripts. The system is capable of outputting metrics such as:

- Estimated library size
- Number of genes/transcripts detected
- Intragenic mapping rates
- Strand-specificity rates
- Correlation matrices to identify similar samples
- Mean coverage of transcripts along transcript lengths

**Figure 8:**
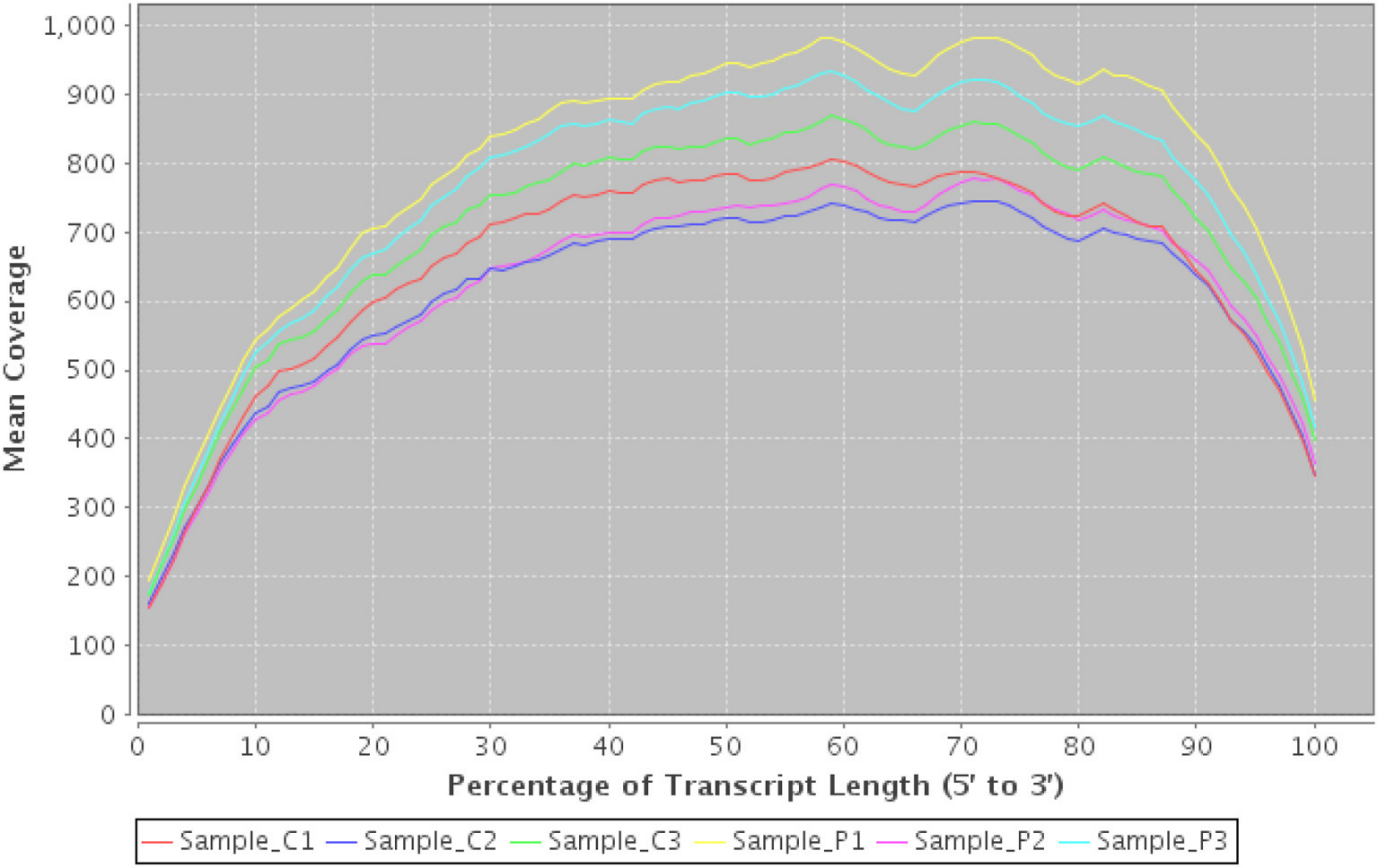
**Transcript coverage using RNASeqQC**. An example plot from RNASeqQC detailing mean coverage of top 20 transcript abundances for 6 samples. A clear bias can be seen at both 3' and 5' ends which may impact on downstream analysis.

These can provide valuable first-pass checks for both the sequencing service and downstream users. Figure 8 illustrates a figure demonstrating a bias of reads toward the start and end of transcripts—possibly caused by polyA extraction. In this case we would conclude that the start of transcripts are likely to be under-represented and that downstream tools such as Cufflinks (Trapnell et al., 2010) may have difficulty reconstructing the start and end of transcripts accurately due to lower coverage.

## Improvements

There are a number of potential improvements to the quality-checks described above.

The ERCC spike-in plots are informative, but at present they require manual review. An automated system to fit and model the goodness of fit would be beneficial. Additionally the ERCC spike-in controls can be used to measure relative abundance *between* samples and can be used to normalize RPKM counts between samples. However, at present no existing software makes use of such spike-in data.

A concern regarding potential bacterial contamination of DNA and RNA extraction kits means that low abundance samples require special care during analysis (Evans et al., 2003; Erlwein et al., 2011). This is of particular concern with low-input library preparations where contaminants may be present at similar abundance to sample material. In particular the transposon based and low-input RNA library preparation methods are at risk when performing metagenomics or metatranscriptomics. The most obvious solution is to run at least one negative control from each kit used for each of the samples. The results of this can be used to eliminate contaminants from any final analysis. The potential for library preparation kits containing contaminants also needs to be investigated as a matter of urgency.

Other tools have also been developed which may prove to be useful quality control tools. These include the Blobology tools (Kumar et al., 2013) to investigate the GC and genome of sequencing libraries. In addition the PAUDA (Huson and Xie, 2014) tool can be used to rapidly identify the taxonomic ID of reads in much the same way as BLAST can be used to classify contigs.

In terms of hardware infrastructure, with Illumina's development of the BaseSpace infrastructure on the Amazon cloud platform, there is also an argument to develop generic “sequencing service infrastructure” platforms on cloud infrastructure. Privacy and data retention policies may not currently permit this for the processing of some samples. However, the economic incentives mean that these issues are likely to be resolved. One could then envisage that a “best-of-breed” infrastructure could be built and deployed for all core facilities, incorporating LIMS, sample tracking, reagent tracking, quality control and data delivery. In that way the collective expertise of the community could be adapted by each facility to best serve its users.

## Feedback

Regardless of which library types are sequenced or how much analysis is performed, one of the most important aspects of providing data is to provide personal feedback to users. If there is a problem with any data generated at a facility, it is important that this is communicated to users at the earliest possible opportunity. It is crucial that this is done regardless of the source of the problem. Data generation should be a partnership between the end-user and the facility generating the data. Despite the decreasing cost of sequencing, biologists often lack the skill and confidence to analyse resulting datasets. The potential for subtle but serious biases affecting downstream analyses requires that sequencing providers undertake an earnest obligation to provide high quality feedback as well as high-quality data.

## Summary

Operation of a sequencer is becoming a relatively routine task for any laboratory with experience of molecular biology. However, the methods involved in sample extraction, library preparation and sequencing are all potentially subject to a variety of biases. The importance of quality control at every step ensures that these biases can be monitored, minimized and enables correction downstream. Most crucially it enables end-users to have confidence in their final results.

### Conflict of interest statement

The authors declare that the research was conducted in the absence of any commercial or financial relationships that could be construed as a potential conflict of interest.
